# Impact of remnant cholesterol on short-term mortality in acute decompensated heart failure: cohort study evidence from Jiangxi, China

**DOI:** 10.3389/fendo.2025.1624112

**Published:** 2025-08-04

**Authors:** Guoan Jian, Kun Jiang, Zihao Lu, Shiming He, Lin Xie, Shuhua Zhang, Qun Wang, Hengcheng Lu, Zhiyu Xiong, Zhiting Wu, Guobo Xie, Guotai Sheng, Aimin Xie, Yanfeng Liu, Wei Wang, Yang Zou

**Affiliations:** ^1^ Jiangxi Medical College, Nanchang University, Nanchang, Jiangxi, China; ^2^ Jiangxi Cardiovascular Research Institute, Jiangxi Provincial People’s Hospital, The First Affiliated Hospital of Nanchang Medical College, Nanchang, Jiangxi, China; ^3^ Department of Cardiology, Jiangxi Provincial People’s Hospital, The First Affiliated Hospital of Nanchang Medical College, Nanchang, Jiangxi, China; ^4^ Department of Cardiovascular Surgery, Jiangxi Provincial People’s Hospital, The First Affiliated Hospital of Nanchang Medical College, Nanchang, China

**Keywords:** remnant cholesterol, acute decompensated heart failure, mortality, longitudinal study, cohort study

## Abstract

**Introduction:**

Remnant cholesterol (RC), a highly atherogenic lipid component, has been strongly implicated in the pathogenesis and adverse clinical outcomes of numerous cardiovascular and cerebrovascular diseases. However, its impact on short-term prognosis in patients with acute decompensated heart failure (ADHF) remains to be elucidated.

**Methods:**

This study enrolled 2,365 patients with acute decompensated heart failure (ADHF) admitted to Jiangxi Provincial People’s Hospital from 2018 to 2024. Participants were stratified into quartiles based on RC. The primary outcome was 30-day all-cause mortality. Multivariable-adjusted Cox regression and restricted cubic spline regression were employed to analyze the association between RC and 30-day mortality in ADHF patients. Additionally, exploratory mediation analyses were performed to assess potential mediating roles of inflammation, oxidative stress, and nutritional factors in this relationship.

**Results:**

During the 30-day follow-up period, 151 deaths were recorded. Mortality was significantly higher in the highest RC quartile compared to the other three groups (Q1:4.41% vs Q2:5.85% vs Q3:5.93% vs Q4:9.18%). After full adjustment for potential confounders, RC demonstrated a significant linear positive association with 30-day mortality in ADHF patients [Hazard ratio: 1.16 (1.05, 1.28)]. Compared with those in the lowest quartile, patients in the highest RC quartile had a 76% increased risk of 30-day mortality. Further subgroup analyses demonstrated that ADHF patients with comorbid hypertension, coronary heart disease, and reduced ejection fraction had a significantly higher 30-day mortality risk than those without these conditions.

**Discussion:**

This Chinese cohort study reveals a dose-dependent relationship between RC and 30-day mortality in ADHF patients, particularly exacerbated in those with hypertension, coronary heart disease, or reduced ejection fraction.

## Graphical Abstract

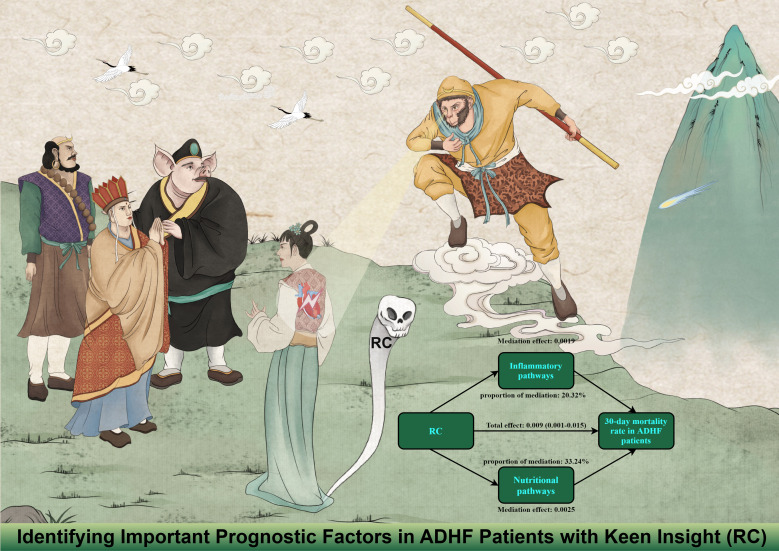

## Background

Heart failure (HF) is a complex clinical syndrome caused by various etiologies, primarily characterized by dyspnea, fatigue, and fluid retention (e.g., pulmonary congestion, systemic venous congestion, and peripheral edema) (1). Epidemiological studies indicate that HF represents the most frequent cause of hospitalization among the elderly population, affecting more than 50 million individuals worldwide (2, 3). With the accelerated progression of global population aging, the overall prevalence of HF is anticipated to increase substantially, thereby imposing a growing economic burden on healthcare systems worldwide (4, 5). Acute decompensated HF (ADHF) is defined as a sudden deterioration or acute exacerbation of chronic or new-onset HF symptoms or signs, typically life-threatening and requiring urgent hospitalization for medical intervention (1, 6). Although HF treatment has improved significantly (7–9), ADHF patients remain at high risk for early readmission and death (10, 11). Evidence indicates that approximately 25% of ADHF patients are readmitted within 30 days post-discharge (12, 13), while mortality rates range from 2.4% to 11.3% (14–18), collectively imposing a substantial disease burden on patients, their families, and healthcare systems.

Atherosclerosis serves as a significant risk factor for various cardiovascular and cerebrovascular diseases, including HF (19–21), with low-density lipoprotein cholesterol (LDL-C) metabolic dysregulation playing a central role in the pathogenesis and progression of atherosclerosis (22, 23). Although statin therapy has been conclusively shown to markedly reduce LDL-C levels, a significant residual cardiovascular risk remains (24, 25). Both observational studies and genetic analyses consistently identify remnant cholesterol (RC) as the predominant contributor underlying this residual risk (26, 27). RC represents the cholesterol component of triglyceride (TG)-rich lipoproteins, which consist of intermediate-density lipoproteins and very-low-density lipoproteins during fasting states, as well as chylomicron remnants in postprandial conditions (28). Prior studies have established that elevated RC levels are strongly associated with multiple metabolic pathologies, including diabetes mellitus and its microvascular complications (29–31), chronic kidney disease (32), fatty liver disease (33, 34), and hypertension (35). Additionally, increased RC concentrations exhibit significant correlations with both the incidence risk of major cardiovascular and cerebrovascular events (36–41) and their subsequent adverse clinical outcomes (42–47). However, existing evidence concerning the prognostic significance of RC in heart failure remains scarce (48–50), and its potential utility for risk stratification in ADHF patients has yet to be established. In the present study, we systematically evaluated the association between RC levels and short-term clinical outcomes in ADHF patients, aiming to provide clinically relevant evidence for early risk stratification in ADHF.

## Methods

### Study population and design

**Figure 1:**
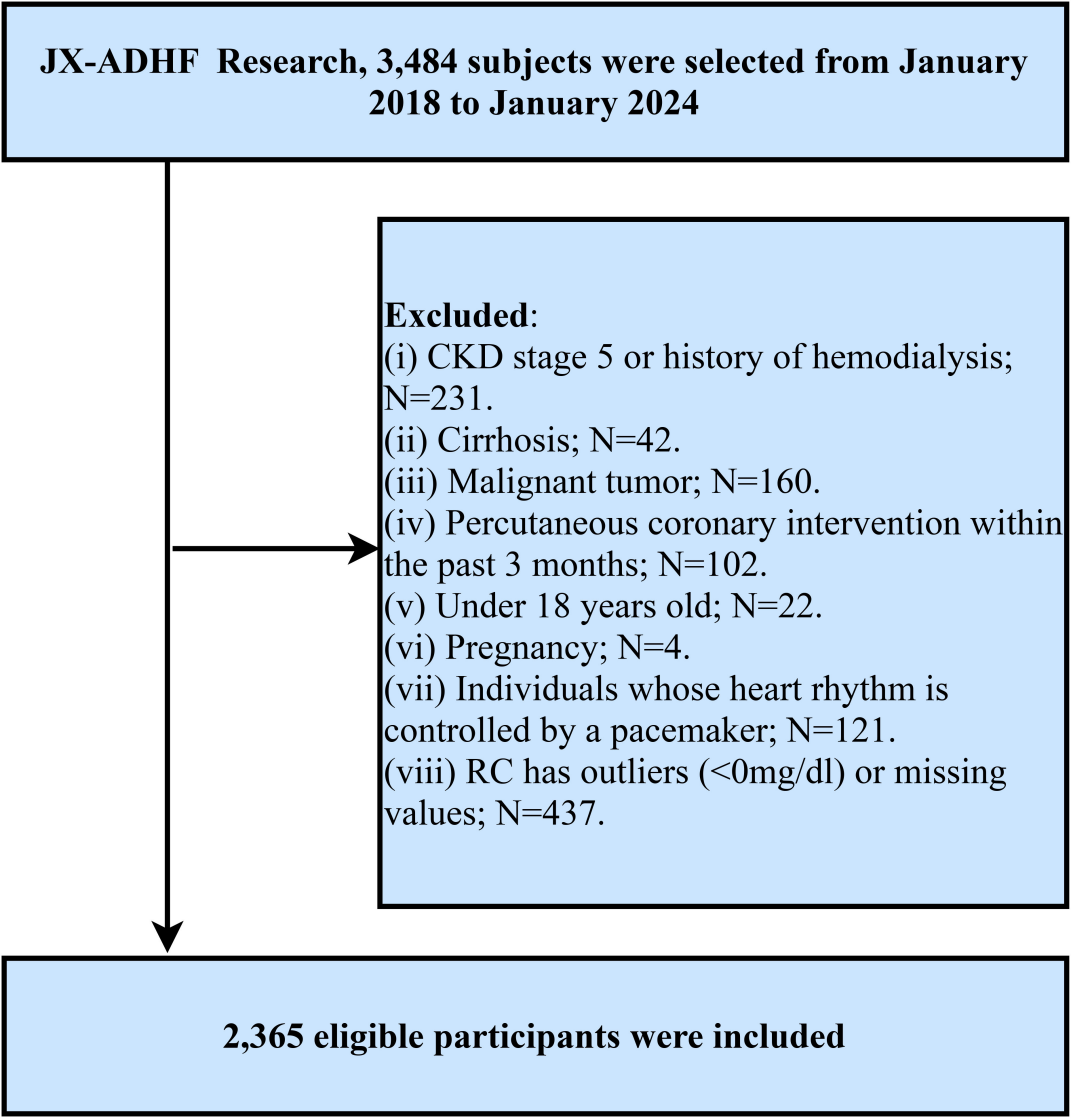
Flow chart of study participants.

The Jiangxi-Acute Decompensated Heart Failure Study II is a hospital-based cohort study initiated by Jiangxi Provincial People’s Hospital, aiming to develop early risk stratification models using clinical data from hospitalized ADHF patients and provide evidence-based strategies to improve outcomes in this high-risk population. Specifically, this study consecutively enrolled 3,484 ADHF patients admitted to Jiangxi Provincial People’s Hospital from January 2018 to January 2024. Subjects with the following characteristics were excluded: (1) To mitigate potential confounding from non-cardiac fluid retention, patients with diagnosed uremia, patients with chronic kidney disease requiring hemodialysis, and patients with liver cirrhosis (n=273) were excluded; (2) Given malignancy’s role as a major survival determinant, we further excluded participants with coexisting malignant tumors (n=160); (3) Since reperfusion therapy plays a significant role in short-term prognosis, patients who had undergone percutaneous coronary intervention within the past 3 months were excluded (n=102); (4) Patients with pacemaker-controlled cardiac rhythms were excluded because their heart rates are not expected to exhibit autonomic nervous system regulation (n=121); (5) Minor participants, referring to those under the age of 18 (n=22), were excluded; (6) Pregnant individuals (n=4) were excluded. Additionally, subjects with missing RC data (n=368) and outliers (n=69) were excluded based on study objectives. Ultimately, 2,365 participants were included in the final analysis. The detailed inclusion and exclusion criteria for the study population are illustrated in **Figure 1**.

### Ethics statement

This retrospective study was conducted in strict compliance with the ethical principles of the Declaration of Helsinki and was approved by the Ethics Review Committee of Jiangxi Provincial People’s Hospital. Informed consent for data utilization was obtained from both patients and their legal representatives [No. 2024 (01)]. The study findings were reported by the Strengthening the Reporting of Observational Studies in Epidemiology guidelines to ensure methodological transparency.

### Data collection

Trained clinical researchers collected the following data from the hospital’s electronic medical record system: demographic information (gender, age), clinical comorbidities [hypertension, diabetes, coronary heart disease (CHD), cerebral infarction], New York Heart Association (NYHA) functional classification, lifestyle habits (smoking status, drinking status), basic measurements [systolic and diastolic blood pressure], and echocardiographic parameters (left ventricular ejection fraction: LVEF). Of note, blood pressure measurements were obtained using an Omron automatic sphygmomanometer (HBP-1300) by medical staff with patients in a rested state (minimum 5-minute rest prior to measurement). Clinical comorbidities were determined based on patients’ medical history records and medication records.

Venous blood samples were collected by trained nurses within 24 hours of patient admission and subsequently sent to the Clinical Laboratory Center of Jiangxi Provincial People’s Hospital for analysis. The measured parameters included red blood cell count (RBC), white blood cell count (WBC), platelet count (PLT), albumin (ALB), alanine aminotransferase, aspartate aminotransferase (AST), gamma-glutamyltransferase (GGT), creatinine (Cr), blood urea nitrogen, uric acid, total cholesterol (TC), TG, LDL-C, high-density lipoprotein cholesterol (HDL-C), fasting plasma glucose, and N-terminal pro-B-type natriuretic peptide. It should be emphasized that all liver enzyme and lipid profile measurements were performed using fasting blood samples collected either at admission or the next morning after an overnight fast.

### Calculation of RC

RC = TC (mg/dl) – HDL-C (mg/dl) - LDL-C (mg/dl) (28).

### Study outcomes

The primary outcome of this study was all-cause mortality within 30 days after admission in ADHF patients. Follow-up period: Patients were followed up starting from the first day after admission until 30 days thereafter. Follow-up methods: Trained clinical researchers conducted non-face-to-face follow-ups through both remote (telephone/text message) and in-person modalities during clinic visits or hospitalizations.

### Handling of missing data

Missing data characteristics are detailed in **Supplementary Table 1**. The proportion of missing covariates was low, with a maximum missingness rate of 4.69%. To maintain the integrity of real-world evidence, all analyses were conducted on the original dataset without imputation.

### Statistical analysis

Participants were stratified into four groups according to RC quartiles. Continuous data following a normal distribution were presented as mean ± standard deviation, while non-normally distributed variables were expressed as median (interquartile range). Categorical measures were described in terms of frequencies and percentages. Intergroup differences were assessed via one-way analysis of variance (normal data), the Kruskal-Wallis test (skewed data), and the chi-square test (categorical comparisons).

Kaplan-Meier survival curves were generated to compare survival rates of ADHF patients in different RC groups, with intergroup differences assessed using the log-rank test. Subsequently, three multivariable Cox proportional hazards regression models with incremental adjustments were constructed to evaluate the association between RC levels and 30-day mortality, expressed as hazard ratios (HRs) with 95% confidence intervals. Model I was adjusted for gender, age, hypertension, diabetes, cerebral infarction, and CHD. Model II was further adjusted for NYHA classification, drinking status, smoking status, LVEF, systolic blood pressure, and diastolic blood pressure. Model III additionally adjusted for WBC, RBC, PLT, ALB, AST, GGT, Cr, blood urea nitrogen, uric acid, HDL-C, LDL-C, fasting plasma glucose, and N-terminal pro-B-type natriuretic peptide in Model II. Before performing these analyses, collinearity was assessed between RC and other covariates (**Supplementary Table 2**) to ensure the exclusion of covariates exhibiting multicollinearity. Furthermore, based on the Schoenfeld residual plot for time-varying RC (**Supplementary Figure 1**), we verified the proportional hazards assumption for the applied models in the current study.

Based on the fully adjusted Model III, we implemented a 4-knot restricted cubic spline to visualize the association between RC levels and 30-day mortality among ADHF patients, and to assess the existence of potential nonlinear relationships.

We further performed subgroup analyses stratified by age (≤70 vs. >70 years), gender (male vs. female), NYHA functional class (III vs. IV), LVEF (<50% vs. ≥50%), and comorbidities (diabetes, hypertension, cerebral infarction, CHD) to evaluate differences in RC-related 30-day mortality. Subgroup heterogeneity was assessed using likelihood ratio tests.

Mediation analysis was conducted to assess the potential mediating roles of oxidative stress, inflammation, and nutritional status in the association between RC and mortality. The proportion mediated was quantified by calculating the ratio of the indirect effect to the total effect. In reference to prior studies, we selected GGT as a marker of oxidative stress (51), WBC as a marker of inflammation (52), and ALB as an indicator of nutritional status (53).

Finally, considering the significant impact of lipid-lowering therapy on RC levels, we conducted a sensitivity analysis: ADHF patients receiving lipid-lowering therapy were excluded, and we further tested the association between RC and 30-day mortality using the same covariate adjustment strategy as previously described.

## Results

### Baseline characteristics of the study population

The cohort consisted of 2,365 ADHF patients, averaging 69 years of age, with a male predominance (1,376, 58.18%) compared to females (989, 41.82%). Based on participants’ medical history and etiology, 252 individuals (10.66%) were classified as new-onset acute HF, while 2,113 (89.34%) were diagnosed with acute decompensation of chronic HF. Baseline characteristics stratified by RC quartiles were presented in **Table 1**. Compared to the low RC group, patients in the high RC group exhibited a significantly higher proportion of females and greater comorbidity burden, with elevated prevalence rates of hypertension, diabetes, cerebral infarction, and CHD; and they also showed higher rates of smoking and drinking status, with a higher proportion of patients classified as NYHA functional class IV. Regarding laboratory parameters, the high RC group exhibited higher SBP, WBC, RBC, PLT, Cr, TC, TG, HDL-C, and LDL-C, while AST and GGT levels were lower.

**Table 1:** Summary of baseline characteristics of the study population according to RC quartile group.

Variable	RC quartiles				*P*-value
	Q1 (0.39-11.60)	Q2 (11.60-17.40)	Q3 (17.79-25.52)	Q4 (25.91-302.01)	
No. of subjects	567	615	573	610	
Age (years)	70.00 (60.00-79.00)	72.00 (62.00-80.00)	72.00 (62.00-80.00)	70.00 (61.00-79.00)	0.072
Gender (n,%)					<0.001
Male	353 (62.26%)	383 (62.28%)	313 (54.62%)	327 (53.61%)	
Female	214 (37.74%)	232 (37.72%)	260 (45.38%)	283 (46.39%)	
NYHA classification (n,%)					0.003
III	406 (71.60%)	431 (70.08%)	388 (67.71%)	379 (62.13%)	
IV	161 (28.40%)	184 (29.92%)	185 (32.29%)	231 (37.87%)	
Hypertension (n,%)					0.010
No	342 (60.32%)	338 (54.96%)	309 (53.93%)	309 (50.66%)	
Yes	225 (39.68%)	277 (45.04%)	264 (46.07%)	301 (49.34%)	
Diabetes (n,%)					<0.001
No	446 (78.66%)	463 (75.28%)	432 (75.39%)	402 (65.90%)	
Yes	121 (21.34%)	152 (24.72%)	141 (24.61%)	208 (34.10%)	
Cerebral infarction (n,%)					0.076
No	486 (85.71%)	514 (83.58%)	470 (82.02%)	489 (80.16%)	
Yes	81 (14.29%)	101 (16.42%)	103 (17.98%)	121 (19.84%)	
CHD (n,%)					0.017
No	407 (71.78%)	406 (66.02%)	382 (66.67%)	385 (63.11%)	
Yes	160 (28.22%)	209 (33.98%)	191 (33.33%)	225 (36.89%)	
Drinking status (n,%)					0.021
No	512 (90.30%)	566 (92.03%)	528 (92.15%)	534 (87.54%)	
Yes	55 (9.70%)	49 (7.97%)	45 (7.85%)	76 (12.46%)	
Smoking status (n,%)					0.003
No	482 (85.01%)	523 (85.04%)	489 (85.34%)	480 (78.69%)	
Yes	85 (14.99%)	92 (14.96%)	84 (14.66%)	130 (21.31%)	
SBP (mmHg)	126.77 (23.76)	127.44 (23.51)	127.56 (24.87)	130.58 (25.49)	0.034
DBP (mmHg)	76.56 (16.19)	76.38 (15.55)	75.60 (15.75)	75.42 (16.23)	0.529
LVEF (%)	46.00 (36.00-55.00)	46.00 (36.00-56.00)	48.00 (36.00-56.00)	47.00 (38.00-56.00)	0.362
WBC (×10^9^/L)	5.90 (4.70-7.60)	5.92 (4.80-7.47)	6.40 (5.00-7.94)	6.57 (5.20-8.80)	<0.001
RBC (×10^12^/L)	4.10 (0.75)	4.02 (0.78)	4.05 (0.75)	4.07 (0.81)	0.391
PLT (×10^9^/L)	157.00 (120.75-202.25)	155.00 (123.00-199.00)	171.00 (126.00-216.00)	175.00 (134.00-228.00)	<0.001
ALB (g/L)	34.78 (4.61)	35.43 (4.57)	35.81 (5.05)	35.96 (5.63)	<0.001
ALT (U/L)	22.00 (14.00-39.00)	21.00 (14.00-36.00)	21.00 (13.00-40.00)	21.00 (14.00-37.00)	0.836
AST (U/L)	27.00 (20.00-38.00)	26.00 (20.00-38.00)	25.50 (19.00-41.75)	25.00 (19.00-37.00)	0.128
GGT (U/L)	44.00 (25.00-85.50)	43.00 (25.00-72.00)	40.50 (24.25-68.75)	39.00 (24.00-71.00)	0.180
Cr (umol/L)	85.00 (67.00-110.00)	88.00 (70.00-122.00)	90.00 (71.50-125.00)	103.00 (75.00-155.00)	<0.001
BUN (mmol/L)	7.04 (5.52-9.43)	7.35 (5.51-10.40)	7.35 (5.58-10.18)	8.09 (6.06-12.33)	<0.001
UA (umol/L)	439.50 (348.75-540.00)	426.00 (343.00-539.50)	410.00 (314.00-532.00)	433.00 (345.00-553.00)	0.063
TC (mg/dL)	134.40 (36.76)	136.83 (32.16)	149.51 (34.61)	174.55 (46.00)	<0.001
TG (mg/dL)	82.40 (67.34-105.43)	92.14 (73.54-116.07)	108.09 (83.28-144.42)	130.24 (97.68-188.50)	<0.001
HDL-C (mg/dL)	37.12 (29.97-43.70)	37.12 (30.16-44.86)	38.28 (31.71-46.40)	38.48 (31.32-48.24)	0.003
LDL-C (mg/dL)	85.46 (66.51-108.47)	80.05 (63.61-101.70)	85.85 (65.35-106.73)	89.71 (71.73-115.53)	<0.001
FPG (mmol/L)	5.20 (4.60-6.00)	5.30 (4.60-6.20)	5.40 (4.70-6.30)	5.40 (4.70-6.60)	<0.001
NT-proBNP (pmol/L)	3645.00 (1976.00-6208.50)	3842.00 (1854.00-6430.00)	3405.00 (1822.00-6147.00)	3585.50 (1659.00-6076.75)	0.096
30-day mortality (n,%)	25 (4.41%)	36 (5.85%)	34 (5.93%)	56 (9.18%)	0.007

CHD, coronary heart disease; NYHA, New York Heart Association; LVEF, left ventricular ejection fraction; SBP, systolic blood pressure; DBP, diastolic blood pressure; TG, triglyceride; TC, total cholesterol; HDL-C, high-density lipoprotein cholesterol; LDL-C, low-density lipid cholesterol; Cr, creatinine; UA, uric acid; WBC, white blood cell count; RBC, red blood cell count; HGB, hemoglobin; PLT, platelet count; ALT, alanine aminotransferase; AST, aspartate aminotransferase; GGT, gamma-glutamyltransferase; ALB, albumin; NT-proBNP, N-Terminal Pro-Brain Natriuretic Peptide; BUN, urea nitrogen; FPG, fasting plasma glucose; RC, remnant cholesterol.

### Follow-up outcomes

**Figure 2:**
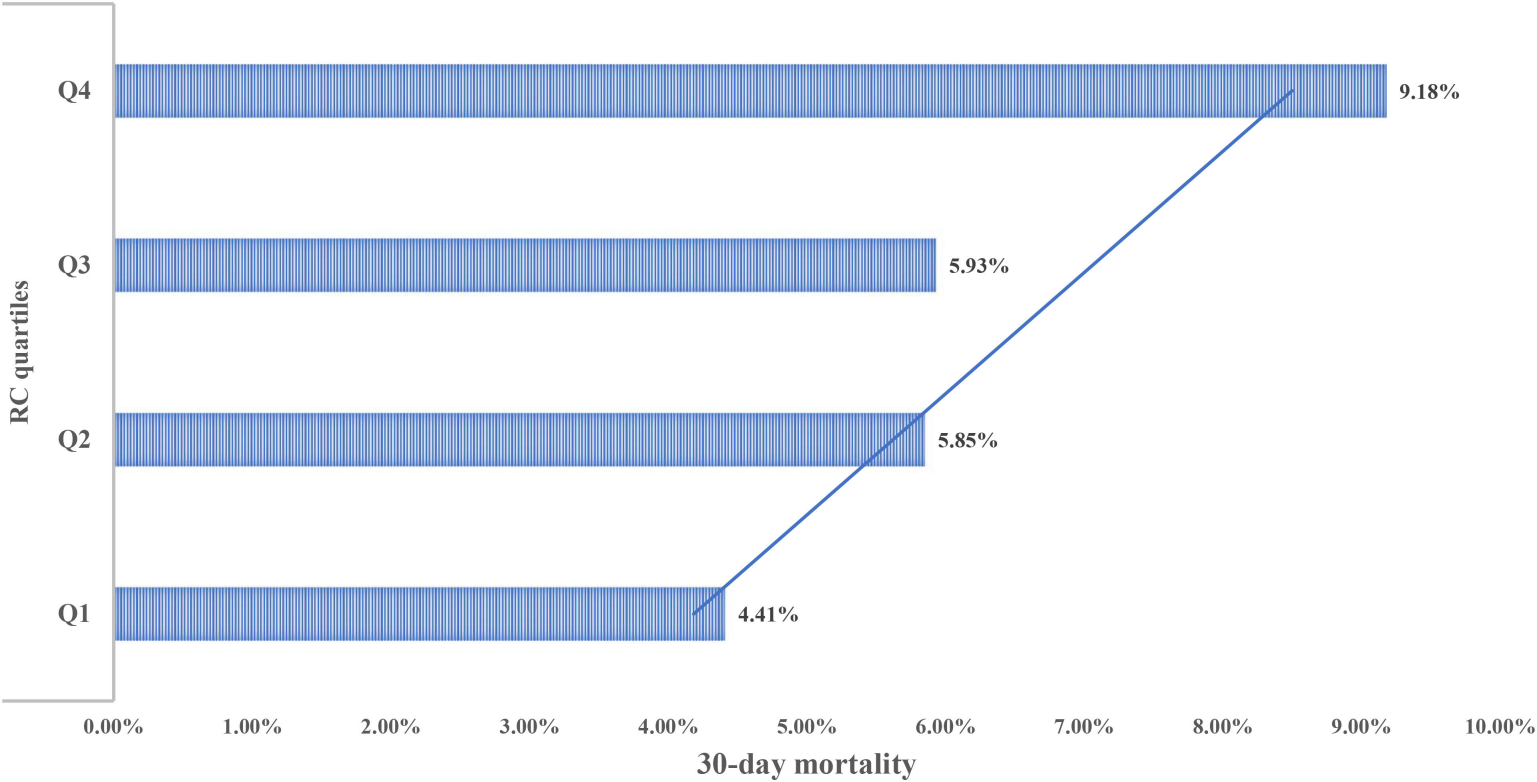
Bar chart showing 30-day mortality of ADHF patients stratified by RC quartiles. RC, Remnant cholesterol; ADHF, acute decompensated heart failure.

After a 30-day follow-up of 2,365 ADHF patients, a total of 151 deaths were recorded. **Figure 2** displays graded increases in 30-day mortality across RC quartiles: Q1: 4.41%, Q2: 5.85%, Q3: 5.93%, and Q4: 9.18%.

**Figure 3:**
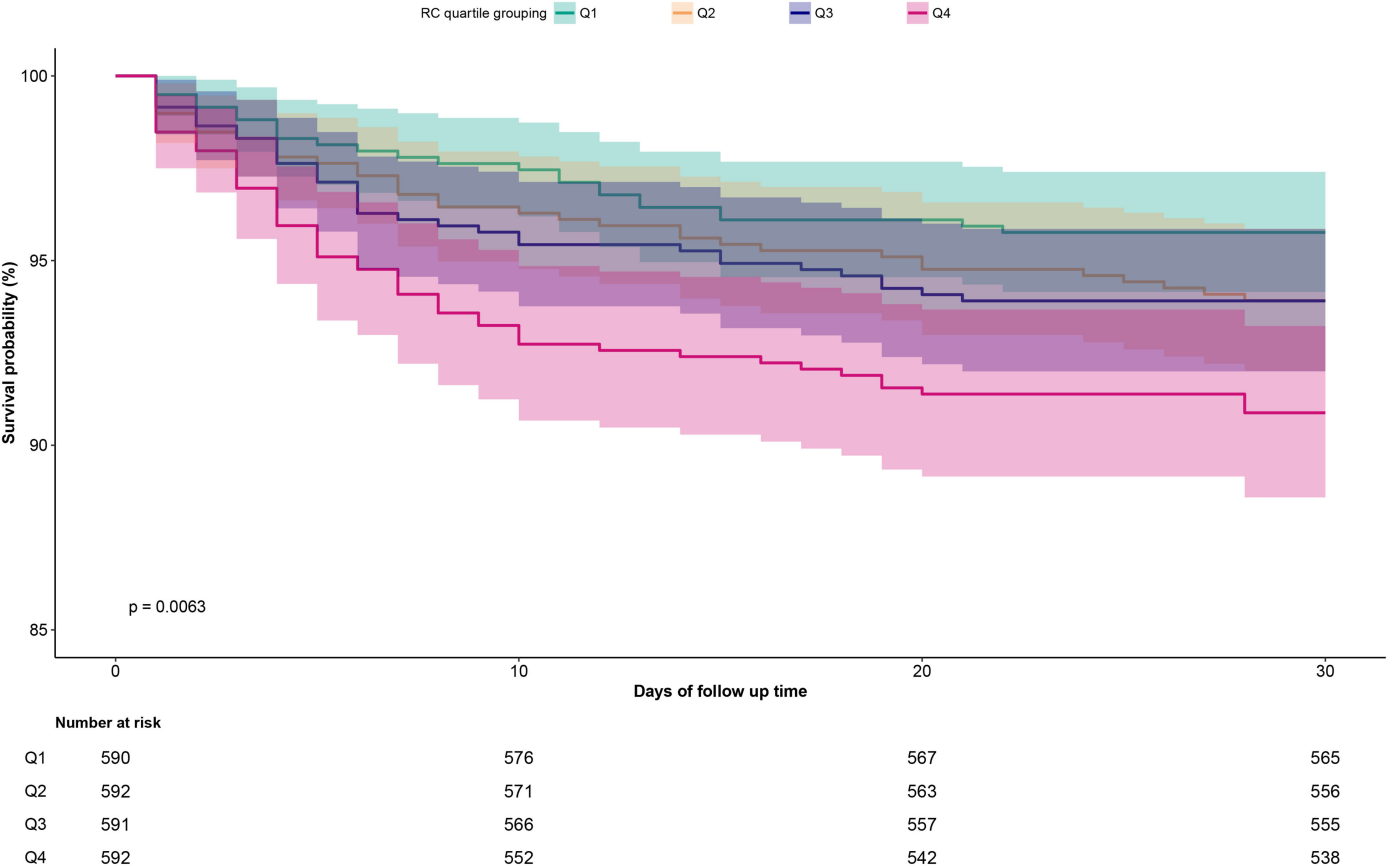
Cumulative survival rate curves of ADHF patients in the RC group. RC, Remnant cholesterol; ADHF, acute decompensated heart failure.

**Figure 3** presents the 30-day survival curves for ADHF patients stratified by RC groups. The highest RC group exhibited a significantly higher 30-day mortality rate compared to the other three groups (Log-rank *p*=0.0063).

### Association of RC with 30-day mortality in ADHF patients

Three progressively adjusted Cox regression models were constructed to analyze the association between RC and 30-day mortality in ADHF patients (**Table 2**). The results showed that RC was significantly positively associated with 30-day mortality in ADHF patients across all models. In the fully adjusted Model III, for every 10 mg/dL increase in RC, the 30-day mortality risk in ADHF patients increased by 16% (HR: 1.16, 95% CI: 1.05, 1.28). Additionally, compared to ADHF patients in the lowest RC group, those in the highest RC group had a 76% increased 30-day mortality risk (HR: 1.76, 95% CI: 1.02, 3.03). Across all models, RC exhibited a significantly positive trend with 30-day mortality in ADHF patients (All *P*-trend < 0.05).

**Table 2:** Multivariable Cox regression analysis of the association between RC and 30-day mortality in patients with ADHF.

Independent variable	Hazard ratios (95% confidence interval)			
	Unadjusted model	Model I	Model II	Model III
RC (Per 10mg/dL increase)	1.11 (1.04, 1.17)	1.11 (1.04, 1.18)	1.13 (1.04, 1.22)	1.16 (1.05, 1.28)
RC (quartiles)				
Q1	Ref	Ref	Ref	Ref
Q2	1.33 (0.80, 2.22)	1.27 (0.76, 2.11)	1.27 (0.74, 2.16)	1.10 (0.63, 1.93)
Q3	1.36 (0.81, 2.28)	1.31 (0.78, 2.20)	1.12 (0.64, 1.95)	1.09 (0.61, 1.94)
Q4	2.14 (1.33, 3.43)	2.02 (1.25, 3.25)	1.75 (1.06, 2.91)	1.76 (1.02, 3.03)
*P*-trend	0.0013	0.0031	0.0352	0.0368

ADHF, acute decompensated heart failure.

Model I adjusted for gender, age, hypertension, diabetes, cerebral infarction and CHD.

Model II adjusted for model I + NYHA classification, drinking status, smoking status, LVEF, SBP, DBP.

Model III adjusted for: Model II + WBC, RBC, PLT, ALB, AST, GGT, Cr, BUN, UA, HDL-C, LDL-C, FPG, NT-proBNP.

**Figure 4:**
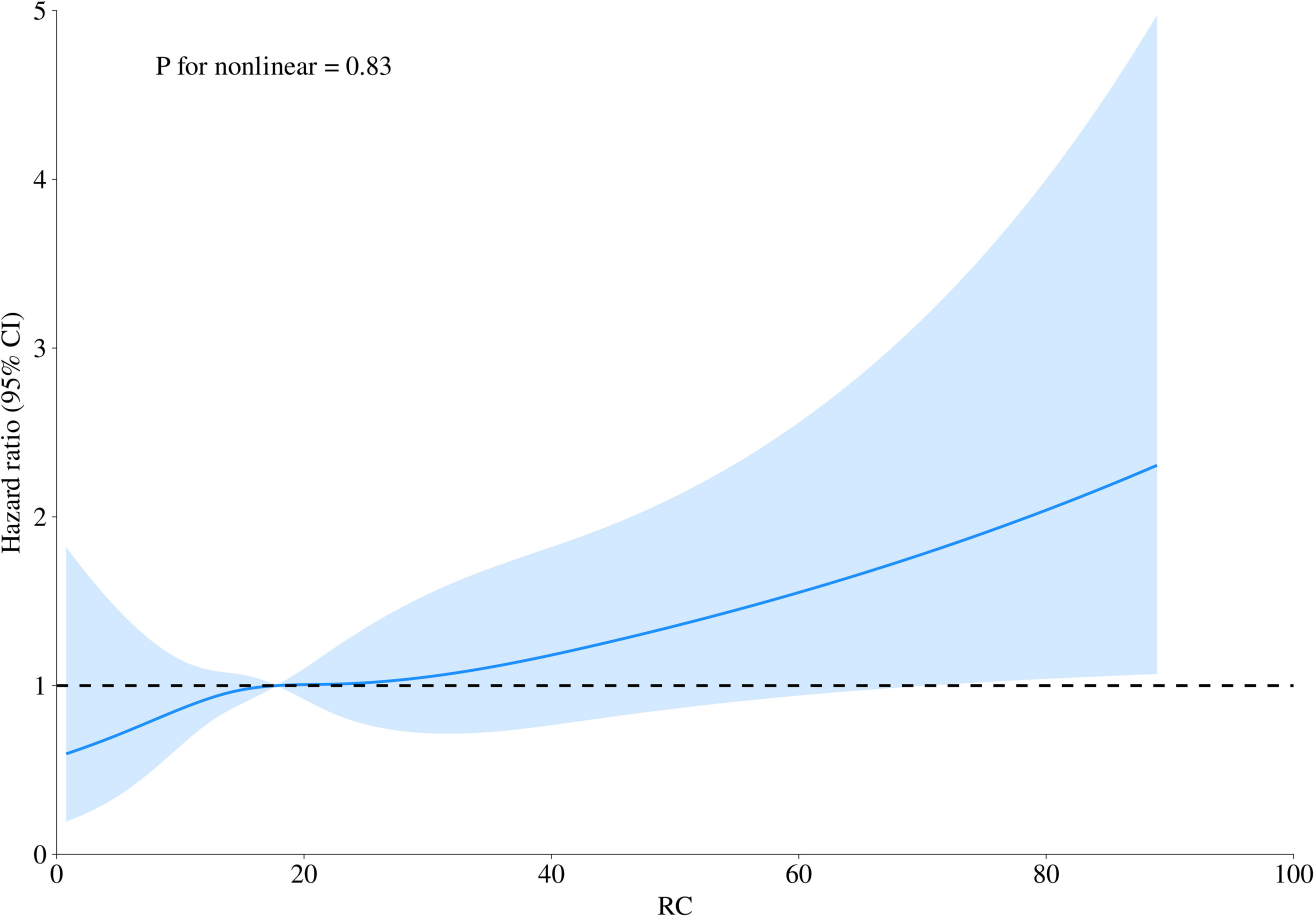
Fitting the dose-response relationship between RC and 30-day mortality in ADHF patients with a 4-knot restricted cubic spline. RC, Remnant cholesterol; ADHF, acute decompensated heart failure. Adjusted for gender, age, hypertension, diabetes, cerebral infarction, CHD, NYHA classification, drinking status, smoking status, LVEF, SBP, DBP, WBC, RBC, PLT, ALB, AST, GGT, Cr, BUN, UA, HDL-C, LDL-C, FPG, NT-proBNP.

Building on adjusted Model III, 4-knot restricted cubic splines were further employed to visualize the dose-response relationship between RC and 30-day mortality in ADHF patients. The results revealed a significant linear positive correlation between RC and 30-day mortality (**Figure 4**, *P* for non-linearity = 0.83).

### Subgroup analysis

Stratified analyses were performed to explore the association between RC and 30-day mortality in ADHF patients according to age, gender, NYHA functional class, LVEF, and clinical comorbidities. The results demonstrated that significant differences in RC-associated 30-day mortality were observed only in the LVEF, hypertension, and CHD subgroups (**Table 3**, *P*-interaction <0.05). Specifically, among hypertensive patients with ADHF, RC-associated 30-day mortality risk was significantly elevated compared to non-hypertensive counterparts (HR: 1.29 vs 0.99). In the CHD subgroup, patients with CHD showed significantly higher RC-associated 30-day mortality risk versus those without (HR: 1.26 vs 1.03). In the LVEF subgroup, patients with LVEF <50% had significantly higher RC-associated 30-day mortality risk than those with LVEF ≥50% (HR: 1.21 vs 0.93).

**Table 3:** Stratified analysis showed the relationship between RC and 30-day mortality in patients with ADHF of different ages, gender, NYHA class, LVEF and whether combined with hypertension/diabetes/cerebral infarction/CHD.

Subgroup	HR Per 10mg/dL increase (95%CI)	*P* for interaction
Age (years)		0.3241
20-70	1.10 (0.91, 1.34)	
71-96	1.22 (1.10, 1.35)	
Gender		0.4197
Male	1.20 (1.10, 1.31)	
Female	1.09 (0.89, 1.35)	
NYHA		0.8030
III	1.17 (1.05, 1.31)	
IV	1.14 (0.98, 1.33)	
LVEF		0.0187
< 50%	1.21 (1.12, 1.31)	
≥ 50%	0.93 (0.74, 1.17)	
Hypertension		0.0046
Yes	1.29 (1.19, 1.41)	
No	0.99 (0.83, 1.18)	
Diabetes		0.6936
Yes	1.12 (0.93, 1.36)	
No	1.17 (1.06, 1.30)	
Cerebral infarction		0.3978
Yes	1.22 (1.08, 1.37)	
No	1.13 (1.00, 1.27)	
CHD		0.0440
Yes	1.26 (1.14, 1.39)	
No	1.03 (0.85, 1.25)	

RC, remnant cholesterol; ADHF, acute decompensated heart failure; CHD, coronary heart disease.

Models adjusted for the same covariates as in model 3 (**Table 3**), except for the stratification variable.

### Mediation analysis

**Figure 5:**
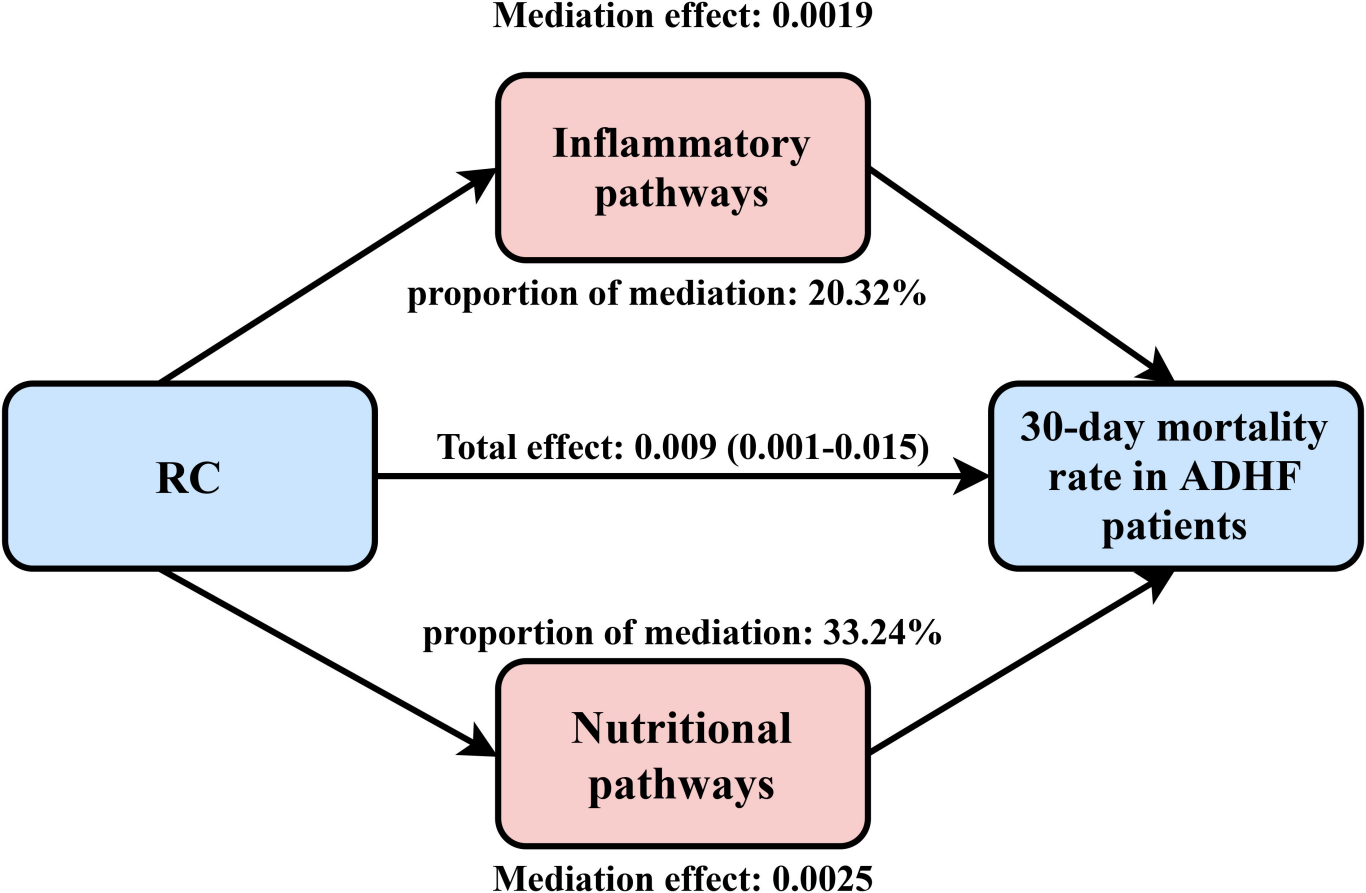
Path diagram for mediational model. RC, Remnant cholesterol; ADHF, acute decompensated heart failure. Adjusted for gender, age, hypertension, diabetes, cerebral infarction, CHD, NYHA classification, drinking status, smoking status, LVEF, SBP, and DBP.

Mediation analysis was conducted to further assess the potential mediating roles of inflammation, oxidative stress, and nutritional factors in the association between RC and 30-day mortality among ADHF patients (**Table 4**). The results revealed that inflammatory factors demonstrated significant mediation in this relationship (*P*-value of mediation proportion = 0.040), contributing to approximately 20.32% of the total effect. Conversely, oxidative stress showed no significant mediating effect (*P*-value of mediation proportion = 0.540). Notably, nutritional factors mediated 33.2% of the effect with marginal significance (**Figure 5**, *P*-value of mediation proportion = 0.080).

**Table 4:** Mediated analysis was performed to explore the roles of inflammation, oxidative stress and nutritional pathways in the association between RC and the 30-day mortality rate in ADHF patients.

Mediator	Total effect	Mediation effect	Direct effect	PM (%)	*P*-value of PM
WBC	0.009 (0.001, 0.015)	0.0019 (0.001, 0.004)	0.007 (0.001, 0.013)	20.32	0.040
GGT	0.009 (0.001, 0.015)	0.0002 (-0.001, 0.005)	0.008 (0.000, 0.015)	2.22	0.540
ALB	0.009 (0.001, 0.015)	0.0025 (0.001, 0.004)	0.007 (0.001, 0.013)	33.24	0.080

RC, remnant cholesterol; PM, proportion mediate; ADHF, acute decompensated heart failure; WBC, white blood cell count; ALB, albumin; GGT, gamma-glutamyltransferase.

The model was adjusted for the same covariates as in model II (**Table 2**), except for the mediator variable.

### Sensitivity analysis

After excluding ADHF patients receiving lipid-lowering therapy, a consistent positive association persisted between RC and 30-day mortality across all models (**Table 5**). Specifically, in unadjusted and adjusted models (Model I-III), each 10 mg/dL increment in RC consistently corresponded to elevated 30-day mortality risk in ADHF patients, with hazard increases of 15%, 13%, 16%, and 22%. Collectively, these findings indicated that the association between RC and 30-day mortality in ADHF patients remained robust and was not significantly confounded by lipid-lowering therapy.

**Table 5:** Multivariable Cox regression analysis of the association between RC and 30-day mortality in ADHF patients without lipid-lowering therapy.

Independent variable	Hazard ratios (95% confidence interval)			
	Unadjusted model	Model I	Model II	Model III
RC (Per 10mg/dL increase)	1.15 (1.08, 1.22)	1.13 (1.06, 1.20)	1.16 (1.06, 1.28)	1.22 (1.08, 1.38)
RC (quartiles)				
Q1	Ref	Ref	Ref	Ref
Q2	1.44 (0.72, 2.88)	1.33 (0.66, 2.65)	1.25 (0.60, 2.62)	1.61 (0.72, 3.63)
Q3	1.48 (0.74, 2.97)	1.31 (0.65, 2.64)	1.16 (0.53, 2.51)	1.27 (0.54, 2.98)
Q4	2.56 (1.35, 4.84)	2.33 (1.22, 4.45)	1.50 (0.73, 3.10)	1.66 (0.73, 3.79)
*P*-trend	0.0039	0.0109	0.3085	0.3400

ADHF, acute decompensated heart failure.

Model I adjusted for gender, age, hypertension, diabetes, cerebral infarction and CHD.

Model II adjusted for model I + NYHA classification, drinking status, smoking status, LVEF, SBP, DBP.

Model III adjusted for: Model II + WBC, RBC, PLT, ALB, AST, GGT, Cr, BUN, UA, HDL-C, LDL-C, FPG, NT-proBNP.

## Discussion

Our findings demonstrate a significant dose-dependent association between RC elevation and 30-day mortality in this ADHF cohort. Subgroup analyses identified substantial heterogeneity across LVEF categories and among hypertensive or CHD subgroups. Specifically, ADHF patients with comorbid hypertension, CHD, or heart failure with reduced ejection fraction exhibited higher RC-associated mortality risks compared to those without hypertension, CHD, and with preserved ejection fraction.

ADHF, a leading cause of hospitalization in the elderly (3), carries high risks of rehospitalization and mortality during the early post-discharge “vulnerable phase” (12–18), after which risks decline sharply (54–56). As a highly atherogenic lipid (28), RC has been consistently linked to both the risk of cardiovascular and cerebrovascular diseases (36–41) and their adverse clinical outcomes (42–46, 49, 50). For instance, regarding HF prognosis, a study of 2,036 ischemic HF patients undergoing percutaneous coronary intervention revealed that high RC levels were associated with a 214% increased mortality risk (49). Similar findings have also been reported in hypertensive HF populations (50). Similarly, in prognostic evaluations of acute coronary syndrome, each standard deviation increments in RC correlated with a 10% to 82.9% increase in mortality risk (42–44). When RC was analyzed as a categorical variable, high-RC group patients showed 96% to 194% elevated risks of adverse cardiovascular events and 49% to 198.1% higher all-cause mortality relative to the low-RC group (42–46). Furthermore, in patients with acute ischemic stroke, elevated RC levels were associated with significantly poorer clinical outcomes. Specifically, compared to patients with low-to-moderate RC levels, the high RC group had a 747% increase in the risk of short-term adverse events, including mortality and early neurological deterioration (47). In the current study, we further elucidated the critical prognostic value of RC in predicting short-term adverse outcomes in ADHF patients. Our analysis demonstrated that each 10 mg/dL increment in RC (as a continuous variable) was associated with a 16% increase in 30-day mortality risk. When analyzed as a categorical variable, ADHF patients in the high-RC group exhibited a 76% greater 30-day mortality risk compared to those in the low-RC group. This finding aligns with previous studies investigating RC’s prognostic value in cardiovascular diseases (42–46). Notably, a recent study from Beijing, China, reported an inverse association between RC and intermediate-term mortality in HF patients (48), which is in contrast to previous studies (49, 50) and our current findings. In contrast to Zhan et al.’s investigation, our study specifically focused on short-term outcomes in ADHF patients, which is a distinction shared by similar prior studies prioritizing intermediate-term endpoints. This fundamental difference in outcome assessment likely explains the observed discrepancies. Prospective studies with long-term follow-up are warranted to validate these findings.

In subgroup analyses, our study identified several high-risk populations: (1) We observed that ADHF patients with hypertension or CHD faced significantly higher RC-associated 30-day mortality risk. Regarding this subgroup-specific finding, we hypothesize—supported by existing literature—that hypertension, CHD, and RC may exert cumulative adverse effects on prognosis in ADHF. Notably, hypertension and CHD represent not only the most prevalent cardiovascular conditions but also critical contributors to atherosclerosis development, functioning both as clinical manifestations and pathogenic drivers of vascular disease: (i) Hypertension accelerates atherosclerosis progression through multiple mechanisms. Firstly, elevated blood pressure increases mechanical stress on vascular walls, promoting the formation and instability of atherosclerotic plaques (57, 58). Moreover, hypertension enhances hematopoiesis, thereby elevating circulating inflammatory cells that exacerbate atherosclerotic inflammation (59). Within the context of hypertension, sympathetic nervous system overactivation represents a critical pathway, not only elevating blood pressure but also modulating hematopoietic activity to drive atherogenesis (59). (ii) Studies indicate that CHD development is typically accompanied by extensive atherosclerotic lesions and significantly increased inflammatory cell recruitment (60). Furthermore, shear stress within coronary artery walls correlates with atherosclerotic plaque progression and arterial remodeling. Low shear stress in coronary segments promotes plaque area expansion and necrotic core growth, whereas high shear stress correlates with necrotic core progression and calcification (61), collectively highlighting the role of hemodynamic forces in atherosclerosis. (iii) On the other hand, elevated RC is closely linked to the risk of hypertension (35) and CHD (36), and may accelerate hypertension progression (35) and worsen clinical outcomes in CHD (62). Thus, atherosclerosis (elevated RC) and hypertension/CHD may interact synergistically to amplify cumulative risk, thereby exacerbating the incidence of adverse events in ADHF patients. (2) Another notable subgroup finding: ADHF patients with reduced ejection fraction demonstrated significantly higher RC-associated 30-day mortality risk compared to those with preserved ejection fraction, suggesting that deterioration in cardiac systolic function further exacerbates the mortality risk linked to elevated RC. Evidence indicates RC exacerbates myocardial ischemia and cardiac dysfunction through multiple pathways, including increased aldosterone synthesis (63–65), promotion of atherosclerosis (66, 67), and induction of inflammatory responses (64, 65). Therefore, in ADHF patients with impaired systolic function, elevated RC often signifies a poorer prognosis. The subgroup analysis results suggest that clinicians should pay attention to the comprehensive management of comorbidities in ADHF patients, emphasizing the evaluation of RC levels. Personalized treatment strategies should be developed based on the patient’s underlying diseases and cardiac functional status, which is of great significance in reducing mortality and improving clinical outcomes.

The underlying mechanism by which elevated RC levels significantly increase 30-day mortality risk in ADHF patients remains unclear, but may be attributable to atherosclerosis-related inflammatory effects. It is well-established that atherosclerosis is a chronic inflammatory disease, where inflammation plays a pivotal role in disease progression, particularly in the transition from early fatty streaks to advanced stenotic lesions (68, 69). Moreover, studies have found that higher RC levels can directly stimulate inflammatory responses and induce the release of pro-inflammatory cytokines such as interleukin-1, interleukin-6, and tumor necrosis factor-α. The cascade reaction of these cytokines can independently contribute to left ventricular dysfunction (70, 71). Based on the above theory and considering the crucial role of inflammation activation in the pathogenesis and progression of acute HF (3, 72), we further explored the potential mediating role of inflammatory factors in the poor prognosis of patients with RC-related ADHF. Using WBC as an inflammatory marker in the mediation analysis model, the results showed that WBC significantly mediated the association between RC and 30-day mortality in patients with ADHF, with a mediation effect accounting for approximately 20.32%. Furthermore, we evaluated the mediating effects of oxidative stress and nutrition status. The results revealed that while oxidative stress showed no significant mediating effect, nutritional status accounted for 33.24% of the association between RC and 30-day mortality in ADHF patients, though this did not reach statistical significance. Based on these findings, we recommend that clinicians assess both inflammation and nutritional status in all ADHF patients with high RC levels.

### Strengths and limitations

The strengths of this study are as follows: (1) This study is the first to reveal a significant positive correlation between RC and 30-day mortality in ADHF patients within the Chinese population; (2) Subgroup analysis results demonstrate that ADHF patients with comorbid hypertension, CHD, or reduced ejection fraction exhibited elevated 30-day mortality risks, suggesting prioritized clinical attention for this high-risk population.

This study also has several limitations: (1) The study population was primarily derived from the Han Chinese population in southern China, necessitating caution when extrapolating the findings to other countries or ethnic groups. (2) As an observational study, establishing causal relationships between RC levels and short-term prognosis in ADHF patients remains challenging, and further research is required to validate these findings. (3) Despite comprehensive adjustment for known risk factors using multivariate Cox regression models, potential unmeasured confounders may remain, introducing unavoidable bias to our findings. (4) It should be noted that the vast majority of participants in this study did not undergo repeated lipid parameter measurements during hospitalization. Therefore, we could only assess the impact of admission RC (single measurement) on short-term outcomes. Subsequent studies, where feasible, are recommended to evaluate the dynamic changes of RC on the prognosis of ADHF.

## Conclusion

This longitudinal study in a Chinese population provides the first evidence demonstrating a significant linear positive association between RC levels and 30-day mortality in ADHF patients. Notably, among ADHF patients with reduced ejection fraction, hypertension, or CHD, the RC-associated 30-day mortality risk was comparatively higher. Based on these findings, we advocate for routine RC monitoring in clinical practice and recommend implementing early risk stratification strategies.

## Data Availability

The raw data supporting the conclusions of this article will be made available by the authors, without undue reservation.
