# Supplementary material for: Impact of remnant cholesterol on short-term mortality in acute decompensated heart failure: cohort study evidence from Jiangxi, China
Source: Front Endocrinol (Lausanne). 2025 Aug 4;16:1624112. doi: 10.3389/fendo.2025.1624112 (PMC12358261; doi:10.3389/fendo.2025.1624112)
Supplement: Supplementary file 1 [file DataSheet1.docx]

Supplementary Table 1. The missing number and rate of covariates.

|  | Non- Missing | Missing |
| --- | --- | --- |
| Gender | 2365 | 0 |
| Age | 2365 | 0 |
| Hypertension | 2365 | 0 |
| Diabetes | 2365 | 0 |
| Cerebral infarction | 2365 | 0 |
| CHD | 2365 | 0 |
| Drinking status | 2365 | 0 |
| Smoking status | 2365 | 0 |
| NYHA classification | 2365 | 0 |
| SBP | 2365 | 0 |
| DBP | 2365 | 0 |
| LVEF | 2254 | 111 |
| WBC | 2342 | 23 |
| RBC | 2342 | 23 |
| PLT | 2342 | 23 |
| ALB | 2346 | 19 |
| ALT | 2346 | 19 |
| AST | 2348 | 17 |
| GGT | 2346 | 19 |
| Cr | 2337 | 28 |
| BUN | 2337 | 28 |
| UA | 2336 | 29 |
| TC | 2365 | 0 |
| TG | 2365 | 0 |
| HDL-C | 2365 | 0 |
| LDL-C | 2365 | 0 |
| FPG | 2264 | 101 |
| NT-proBNP | 2365 | 0 |
| RC | 2365 | 0 |

Abbreviations as in Table 1.

Supplementary Table 2: Collinearity diagnostics steps.

|  | VIF | | |
| --- | --- | --- | --- |
|  | Step 1 | Step 2 | Step 3 |
| RC | 100022920.9 | 1.8 | 1.8 |
| Gender | 1.3 | 1.3 | 1.3 |
| Age | 1.5 | 1.5 | 1.5 |
| Hypertension | 1.3 | 1.3 | 1.3 |
| Diabetes | 1.4 | 1.4 | 1.4 |
| Cerebral infarction | 1.1 | 1.1 | 1.1 |
| CHD | 1.2 | 1.2 | 1.2 |
| Drinking status | 1.5 | 1.5 | 1.5 |
| Smoking status | 1.5 | 1.5 | 1.5 |
| NYHA classification | 1.1 | 1.1 | 1.1 |
| SBP | 1.8 | 1.8 | 1.8 |
| DBP | 1.6 | 1.6 | 1.6 |
| LVEF | 1.3 | 1.3 | 1.3 |
| WBC | 1.4 | 1.4 | 1.4 |
| RBC | 1.5 | 1.5 | 1.5 |
| PLT | 1.3 | 1.3 | 1.3 |
| ALB | 1.3 | 1.3 | 1.3 |
| ALT | 7.8 | 7.8 | NA |
| AST | 7.5 | 7.5 | 1.1 |
| GGT | 1.2 | 1.2 | 1.1 |
| Cr | 2.5 | 2.5 | 2.5 |
| BUN | 2.9 | 2.9 | 2.9 |
| UA | 1.6 | 1.6 | 1.6 |
| TC | 681555833 | NA | NA |
| TG | 2 | 2 | 2 |
| HDL-C | 55665542.3 | 1.4 | 1.4 |
| LDL-C | 429059231.4 | 1.2 | 1.2 |
| FPG | 1.4 | 1.4 | 1.4 |
| NT-proBNP | 1.3 | 1.3 | 1.3 |

VIF: variance inflation factor; VIF = 1/(1-R^2^). Abbreviations as in Table ​1.

Note: The variables with VIF>5 will be regarded as collinear variables and cannot be included in the multiple regression model.
