# Supplementary material for: Impact of remnant cholesterol on short-term mortality in acute decompensated heart failure: cohort study evidence from Jiangxi, China
Source: Front Endocrinol (Lausanne). 2025 Aug 4;16:1624112. doi: 10.3389/fendo.2025.1624112 (PMC12358261; doi:10.3389/fendo.2025.1624112)
Supplement: Supplementary file 2 [file DataSheet2.docx]

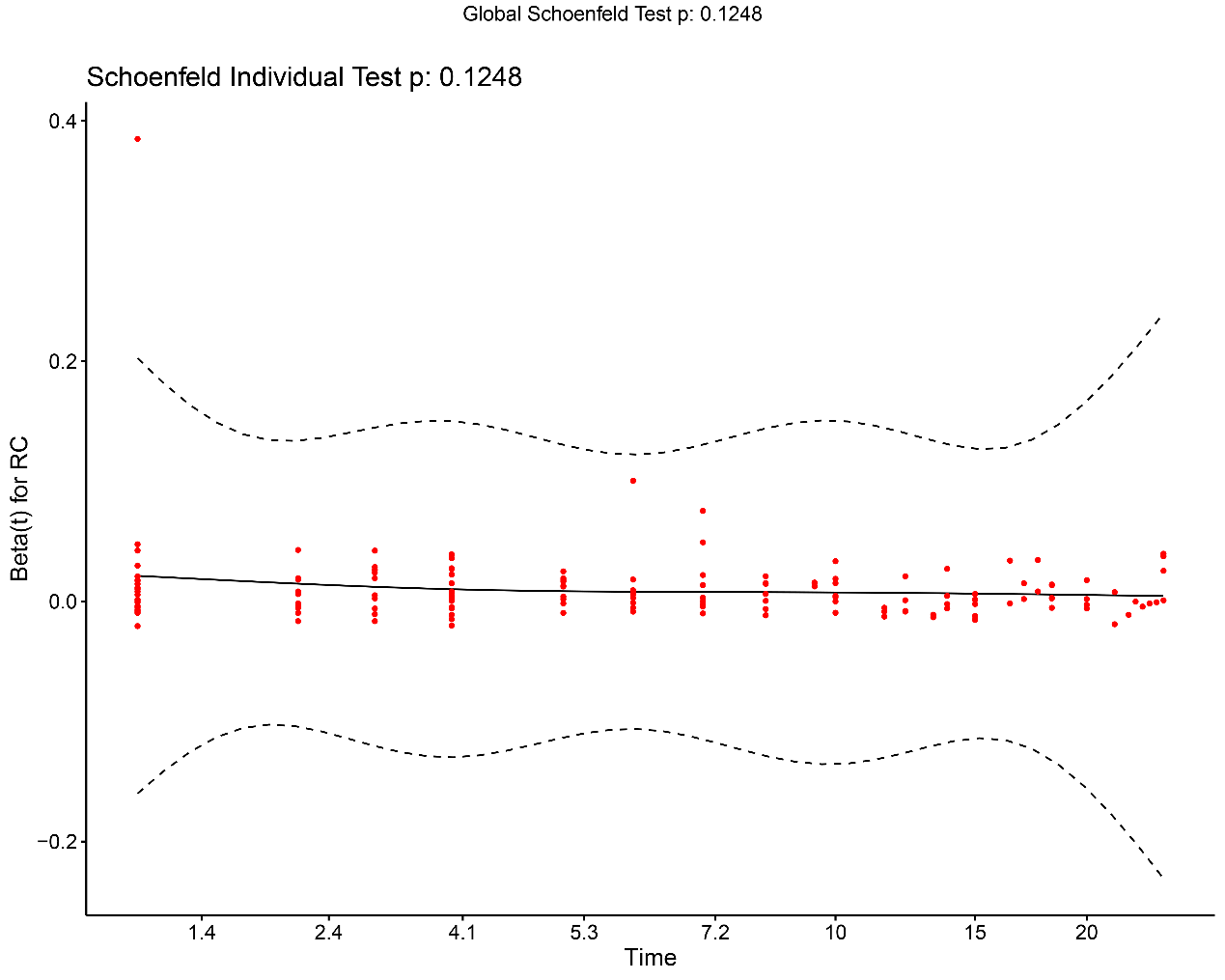


**Supplementary Figure 1**: Schoenfeld residual plot of RC over time with 30-day mortality in ADHF patients as the dependent variable. The p-value of Schoenfeld Residuals Test result is larger than 0.05 which indicated that RC is not a time dependent variable and can be analyzed by Cox Proportional Hazards Model.
